# Tumor infiltrating lymphocyte signature is associated with single nucleotide polymorphisms and predicts survival in esophageal squamous cell carcinoma patients

**DOI:** 10.18632/aging.202798

**Published:** 2021-04-04

**Authors:** Chen Suo, Huiyao Chen, Franciszek Binczyk, Renjia Zhao, Jiahui Fan, Xiaorong Yang, Ziyu Yuan, David Kreil, Paweł Łabaj, Tiejun Zhang, Ming Lu, Li Jin, Joanna Polańska, Xingdong Chen, Weimin Ye

**Affiliations:** 1 Department of Epidemiology and Ministry of Education Key Laboratory of Public Health Safety, School of Public Health, Fudan University, Shanghai, China; 2 Fudan University Taizhou Institute of Health Sciences, Taizhou, China; 3 Center for Molecular Medicine of Children's Hospital of Fudan University, Institutes of Biomedical Sciences, Fudan University, Shanghai, China; 4 Silesian University of Technology, Data Mining Division, Gliwice, Poland; 5 State Key Laboratory of Genetic Engineering and Collaborative Innovation Center for Genetics and Development, School of Life Sciences, Fudan University, Shanghai, China; 6 Clinical Epidemiology Unit, Qilu Hospital of Shandong University, Jinan, China; 7 IMBT Bioinformatics Research, Boku University Vienn, Vienna, Austria; 8 Malopolska Centre of Biotechnology, Jagiellonian University, Krakow, Poland; 9 Human Phenome Institute, Fudan University, Shanghai, China; 10 Department of Medical Epidemiology and Biostatistics, Karolinska Institute, Stockholm, Sweden

**Keywords:** esophageal squamous cell cancer, tumor infiltrating lymphocytes, single nucleotide polymorphism, survival

## Abstract

Purpose: Esophageal cancer is the sixth leading cause of cancer-related death worldwide, and is associated with a poor prognosis. Stromal tumor infiltrating lymphocytes (sTIL) and certain single nucleotide polymorphisms (SNPs) have been found to be predictive of patient survival. In this study, we explored the association between SNPs and sTIL regarding the predictability of disease-free survival in patients with esophageal squamous cell carcinoma (ESCC).

Materials and methods: We collected 969 pathologically confirmed ESCC patients from 2010 to 2013 and genotyped 101 SNPs from 59 genes. The number of sTIL for each patient was determined using an automatic algorithm. A Kruskal-Wallis test was used to determine the association between genotype and sTIL. The genotypes and clinical factors related to survival were analyzed using a Kaplan-Meier curve, Cox proportional hazards model, and log-rank test.

Results: The median age of the patients was 67 (42-85 years), there was a median follow-up of 851.5 days and 586 patients died. The univariable analysis showed that 10 of the 101 SNPs were associated with sTIL. Six SNPs were also associated with disease-free survival. A multivariable analysis revealed that sTIL, rs1801131, rs25487, and rs8030672 were independent prognostic markers for ESCC patients. The model combining SNPs, clinical characteristics and sTIL outperformed the model with clinical characteristics alone for predicting outcomes in ESCC patients.

Conclusion: We discovered 10 SNPs associated with sTIL in ESCC and we built a model of sTIL, SNPs and clinical characteristics with improved prediction of survival in ESCC patients.

## INTRODUCTION

Esophageal cancer (EC) is the seventh most common malignant neoplasm and ranks sixth among cancer-related deaths worldwide [1]. Eastern Asia has the highest incidence rate of EC [1]. It is estimated that there were more than 570,000 newly diagnosed EC cases, approximately 508,000 deaths due to EC in 2018 [1]. EC can be divided into esophageal squamous cell carcinoma (ESCC) and adenocarcinoma based on histopathological classification, with ESCC accounting for about 90% of EC cases [2]. Since it is associated with mild symptoms, ESCC is often diagnosed only after the disease has progressed to a late stage, often meaning that the tumor cannot be resected or cured [3]. Therefore, the five-year survival rate in developed countries is less than 20% [3, 4]. The main factors affecting the prognosis of ESCC include the treatment method, genetic markers, and the general health of the patient.

Tumor infiltrating lymphocytes (TILs), have been shown to reflect the status of the tumor immune microenvironment [5] and are widely considered to be a key indicator of host-tumor immune interactions. Moreover, TILs are predictive biomarkers of potentially effective anti-tumor therapies, including cancer immunogenicity, clinical outcome, and immunotherapy response [6–11]. Recently, many clinical studies have focused on treatments targeting the tumor immune microenvironment and have evaluated the outcome of TILs in several cancers, such as breast cancer and lung cancer [12, 13]. It has been shown that high levels of TILs provide a significant survival advantage in ESCC [14]. Such TILs consist of various lymphocytes (e.g., CD8+, CD4+ and Foxp3+ T cells), which are involved in determining tumor progression and aggressiveness [15, 16]. In addition, stromal tumor infiltrating lymphocytes (sTIL) are defined as lymphocytes that do not directly interact with the tumor cells and are more sensitive at predicting a response to therapy than intratumoural TIL (iTIL) [13].

Both the risk of developing ESCC and patient prognosis may be influenced by genetic variations, (e.g., polymorphisms) [17, 18], which are involved in many cellular pathways, including cell proliferation, DNA repair, apoptosis, and the immune response [19]. Although both patient genetic variation and immune status contribute to the clinical outcome, whether the association between sTIL and ESCC survival varies with different genetic backgrounds unknown. A better understanding of the relationship between genotypes, sTIL, and their combined-impact on the prognosis of ESCC patients may also help to enhance the ability to predict patient survival and promote individualized treatment. Furthermore, most TILs values reported in previous studies have been manually estimated by pathologist, and thus may be subject to interobserver variability.

Although significant progress has been made in understanding the risk factors for esophageal cancer, the development of anti-cancer therapies that can significantly extend the life expectancy of patients has proven challenging [20]. Therefore, we emphasis on studying the patterns of patient clinical outcomes and its possible determinants, such as germline mutations and immunological response. In this study, we aimed to investigate the relationship between SNPs and automatically quantified sTIL, we also sought to explore their impact on the prognosis of ESCC in Taixing, China, a medium sized city in eastern China, with the highest incidence rate of ESCC.

## RESULTS

### sTIL characteristics

The patient demographic characteristics are listed in Table 1. The median age at diagnosis was 67 years (range: 42 – 85 years) and the patients were predominately male (66.6%). Of all patients, 48.3% (468/965) of the cases were first treated with surgery, 12.4% (120/965) by chemotherapy and 12.1% (117/965) by radiotherapy. The percentage sTIL was associated with the first-line treatment method (*P* = 0.006). A borderline significant association was observed for tea drinking (*P* = 0.068).

**Table 1:** Clinicopathological factors and association with sTIL in ESCC.

**Clinical character**	**Count(%)**	**sTIL(95% CI)**	***P* value**
Age grouping			0.278
40-49	28 (2.9)	27.84 (25.57, 32.63)	
50-59	156 (16.1)	28.50 (23.70, 37.00)	
60-69	410 (42.3)	28.62 (22.03, 35.76)	
70-79	312 (32.2)	27.18 (21.37, 33.95)	
80-85	63 (6.5)	29.27 (23.31, 36.04)	
Sex			0.515
Male	645 (66.6)	28.22 (22.77, 34.91)	
Female	324 (33.4)	28.23 (21.84, 36.28)	
First-degree family history of ESCC			0.804
No	640 (66.0)	28.37 (22.40, 35.53)	
Yes	299 (30.9)	28.07 (22.44, 35.26)	
Missing	30 (3.1)	NA	
Grade of Differentiation			0.246
Gx Grading cannot be evaluated	49 (5.1)	29.24 (23.69, 35.60)	
G1 Highly differentiated	72 (7.4)	31.38 (23.77, 36.90)	
G2 Medium differentiation	563 (58.1)	28.22 (22.30, 35.18)	
G3 Poorly differentiated	181 (18.7)	28.80 (22.15, 36.18)	
G4 Undifferentiated	63 (6.5)	25.19 (20.35, 33.41)	
Missing	41 (4.2)	NA	
First-line treatment method			0.006
Surgery	468 (48.3)	28.93 (23.13, 36.31)	
Chemotherapy	120 (12.4)	25.73 (19.97, 32.49)	
Radiotherapy	117 (12.1)	27.69 (22.71, 33.90)	
Combination therapy	221 (22.8)	28.89 (21.84, 35.60)	
Untreated	39 (4.0)	23.59 (17.95, 33.13)	
Missing	4 (0.4)	NA	
BMI			0.504
<18.5	102 (10.5)	26.45 (20.88, 34.52)	
[18.5,24)	619 (63.9)	28.19 (22.68, 35.49)	
[24,28)	219 (22.6)	28.69 (21.98, 35.47)	
≥28	29 (3.0)	30.34 (24.43, 36.24)	
Smoking(pack-years)			0.940
Never	400 (41.3)	27.90 (21.80, 36.03)	
<30	224 (23.1)	28.09 (23.10, 34.00)	
≥30	304 (31.4)	28.73 (22.76, 35.48)	
Missing	41 (4.2)	NA	
Alcohol consumption status			0.341
No	441 (45.5)	27.59 (21.67, 35.44)	
Ex-drinker	25 (2.6)	28.22 (23.13, 33.68)	
Current drinker	464 (47.9)	29.05 (23.22, 35.54)	
Missing	39 (4.0)	NA	
Tea drinking			0.068
No	628 (64.8)	27.94 (21.85, 35.04)	
Yes	303 (31.3)	29.18 (23.27, 36.63)	
Missing	38 (3.9)	NA	
Times of tooth brushing daily			0.688
1	774 (79.9)	28.21 (22.08, 35.54)	
≥2	169 (17.4)	28.79 (23.45, 34.91)	
Missing	26 (2.7)	NA	
Wealth score			0.178
Q1	299 (30.9)	28.37 (22.99, 35.46)	
Q2	181 (18.7)	26.34 (21.27, 34.08)	
Q3	221 (22.8)	27.73 (23.07, 34.38)	
Q4	170 (17.5)	30.40 (23.01, 37.08)	
Q5	98 (10.1)	26.23 (21.27, 37.47)	
Education level			0.147
Illiteracy	360 (37.2)	27.38 (21.30, 34.87)	
Primary school	368 (38.0)	28.22 (22.94, 35.48)	
Junior high school	186 (19.2)	29.25 (23.41, 35.98)	
High school and above	55 (5.7)	30.33 (24.62, 38.02)	

sTIL, stromal infiltrating lymphocytes.

### SNPs associated with the percentage of sTIL

Of the 1,190 genotyped eligible cases, 73 (6.1%) were lost to follow-up and 148 patients had low image quality of the tissue slides. Therefore, a prognostic association study was carried out for a total of 969 patients. Among 101 SNPs, 10 were found to be associated with the percentage of sTIL (Table 2) based on a Kruskal-Wallis H test. Patients with rs1799930 genotype A/A had the highest percentage of sTIL among all the SNPs. For each SNP, pairwise within-group comparisons were performed to investigate which genotypes were associated with a significantly high percentage of sTIL. The percentage of sTIL in patients with the rs1800682 genotype T/C was significantly higher than that of patients carrying the genotypes T/T and C/C (P < 0.01). The rs2234767 genotype G/A was significantly higher than that of patients carrying A/A. Similarly, for the rs1312200, rs2051428 and rs3762894 polymorphisms, heterozygous patients had a higher percentage of sTIL than the homozygous patients (Supplementary Figure 1).

**Table 2:** Significant SNPs (P-values < 0.05) from a Kruskal-Wallis test for the association with ESCC (N=969).

**SNP**	**Position**	**Gene (or the nearest)**	**A/B alleles**	**# of patients** **(%)**	**sTIL, mean (95% CI)**	***P*-value**
rs1051740	chr1:225831932	EPHX1	C/C	195 (20.1)	28.31 (23.45, 33.93)	0.037
			T/C	472 (48.7)	28.81 (22.92, 36.72)	
			T/T	295 (30.4)	27.01 (20.71, 34.22)	
			Missing	7 (0.7)		
rs1312200	chr4:99091206	LOC100507053	C/C	443 (45.7)	27.23 (21.51, 34.04)	0.001
			T/T	409 (42.2)	29.90 (23.31, 36.90)	
			C/T	107 (11.0)	26.32 (22.28, 33.22)	
			Missing	10 (1.0)		
rs3762894	chr4:99144933	ADH4	C/C	437 (45.1)	27.37 (21.47, 34.08)	0.002
		LOC100507053	C/T	406 (41.9)	29.66 (23.51, 36.76)	
			T/T	107 (11.0)	26.39 (22.39, 33.33)	
			Missing	19 (2.0)		
rs2051428	chr4:99202029	LOC100507053	C/C	496 (51.2)	27.53 (21.61, 34.09)	0.004
			C/T	382 (39.4)	29.61 (23.39, 36.90)	
			T/T	85 (8.8)	26.32 (22.17, 33.24)	
			Missing	6 (0.6)		
rs10008281	chr4:99221145	ADH6	A/A	515 (53.1)	27.59 (21.64, 34.27)	0.009
			A/C	368 (38.0)	29.58 (23.72, 36.92)	
			C/C	77 (7.9)	26.95 (22.17, 33.42)	
			Missing	9 (0.9)		
rs10052657	chr5:59111944	PDE4D	A/A	15 (1.5)	21.67 (17.12, 24.88)	0.008
			C/C	212 (21.9)	28.55 (23.09, 34.13)	
			C/A	720 (74.3)	28.27 (22.43, 35.80)	
			Missing	22 (2.3)		
rs2285947	chr7:21544470	DNAH11	A/A	73 (7.5)	24.32 (19.23, 32.05)	0.021
			G/A	391 (40.4)	28.80 (22.63, 36.31)	
			G/G	474 (48.9)	28.06 (22.67, 35.00)	
			Missing	31 (3.2)		
rs1799930	chr8:18400593	NAT2	A/A	41 (4.2)	31.55 (24.49, 36.96)	0.021
			G/A	334 (34.5)	29.09 (23.17, 36.87)	
			G/G	554 (57.2)	27.36 (21.75, 34.07)	
			Missing	40 (4.1)		
rs1800682	chr10:88990206	FAS	C/C	127 (13.1)	27.41 (19.99, 34.01)	0.015
		ACTA2	T/C	474 (48.9)	29.09 (23.14, 36.41)	
			T/T	348 (35.9)	27.43 (21.67, 34.31)	
			Missing	20 (2.1)		
rs2234767	chr10:88989499	FAS	A/A	108 (11.1)	26.93 (19.57, 33.65)	0.030
		ACTA2	G/A	444 (45.8)	28.73 (23.14, 36.09)	
			G/G	401 (41.4)	27.76 (22.15, 35.03)	
			Missing	16 (1.7)		

### Prognostic value of sTIL and SNPs

Next, we examined the prognostic value of the sTIL and SNPs. The six SNPs that were significantly associated with patient prognosis are presented in Table 3. Because the DFS of patients receiving chemotherapy, chemoradiotherapy and surgery is significantly different. Therefore, if the overall analysis of these three populations is performed without distinction, the difference caused by SNPs may be masked. We add a stratification analysis by the first-line treatment, the six SNPs had significant or borderline significant differences in prognosis in at least one of the four first-line treatment method except for rs994771 (Supplementary Table 1). Patients with rs1801131 C/C had a higher risk of death than those with A/A (Adjusted HR (aHR) = 1.869; 95% CI = 1.183 – 2.954; P = 0.007) and the results changed little after adjusting for staging information (aHR = 1.947; 95% CI = 1.098 – 3.453; P = 0.023) (Supplementary Table 2). Patients with the rs8030672 T/T genotype had a higher risk of death than those carrying the T/A genotype (aHR = 1.545; 95% CI = 1.045 – 2.284, P = 0.029). Patients carrying the rs25487 G/G genotype exhibited better survival compared with the A/A genotype (aHR = 0.619; 95% CI = 0.424 – 0.906; P = 0.014). The percentage of sTIL among the rs994771, rs2234767, and rs1800682 genotypes also significantly differed (Table 3), however, this significance was lost after adjusting for sTIL, age, gender, grade of differentiation, first-line treatment method, and BMI.

**Table 3:** Univariate and multivariate cox regression analyses of SNPs for DFS (N=969).

**SNP**	**Position**	**Gene (or the nearest)**	**A/B alleles**	**Count**	**Median survival (days)**	**Univariate analysis**			**Multivariate analysis**	
						***P*-value**	**HR (95% CI)**		***P*-value**	**HR (95% CI)**
rs1801131	chr1:11794419	MTHFR	A/A	632	1003	0.018	Ref.			
			A/C	256	744		1.238(1.030-1.488)		0.151	1.151(0.950-1.394) ^a^
			C/C	30	649		1.537(1.008-2.343)		**0.007**	1.869(1.183-2.954) ^a^
rs994771	chr4:99406646	LOC102723576	C/C	106	667	0.048	Ref.			
			T/C	385	909		0.779(0.600-1.012)		0.079	0.781(0.594-1.029) ^a^
			T/T	458	993		0.723(0.559-0.936)		0.065	0.776(0.593-1.016) ^a^
rs2234767	chr10:88989499	FAS	A/A	108	776	0.004	Ref.			
		ACTA2	G/A	444	1172		0.771(0.591-1.007)		0.052	0.761(0.578-1.002) ^b^
			G/G	401	791		1.024(0.787-1.333)		0.850	0.974(0.742-1.279) ^b^
rs1800682	chr10:88990206	FAS	C/C	127	899	0.023	Ref.			
		ACTA2	T/C	474	1037		0.860(0.668-1.107)		0.159	0.828(0.638-1.076) ^b^
			T/T	348	790		1.100(0.851-1.422)		0.958	1.007(0.772-1.315) ^b^
rs8030672	chr15:68766745	ANP32A	T/A	62	1734	0.018	Ref.			
			T/T	905	883		1.596(1.085-2.350)		**0.029**	1.545(1.045-2.284) ^a^
rs25487	chr19:43551574	XRCC1	A/A	43	783	0.001	Ref.			
			G/A	354	700		0.948(0.651-1.379)		0.632	0.910(0.619-1.338) ^a^
			G/G	553	1078		0.704(0.487-1.019)		**0.014**	0.619(0.424-0.906) ^a^

Abbreviations: DFS, disease-free survival; CI, confidence interval; HR, hazard ratio.

^a^with adjustment for sTIL, age, sex, grade of differentiation, first-line treatment method, BMI.

^b^with adjustment for age, sex, grade of differentiation, first-line treatment method, BMI.

**Figure 1:**
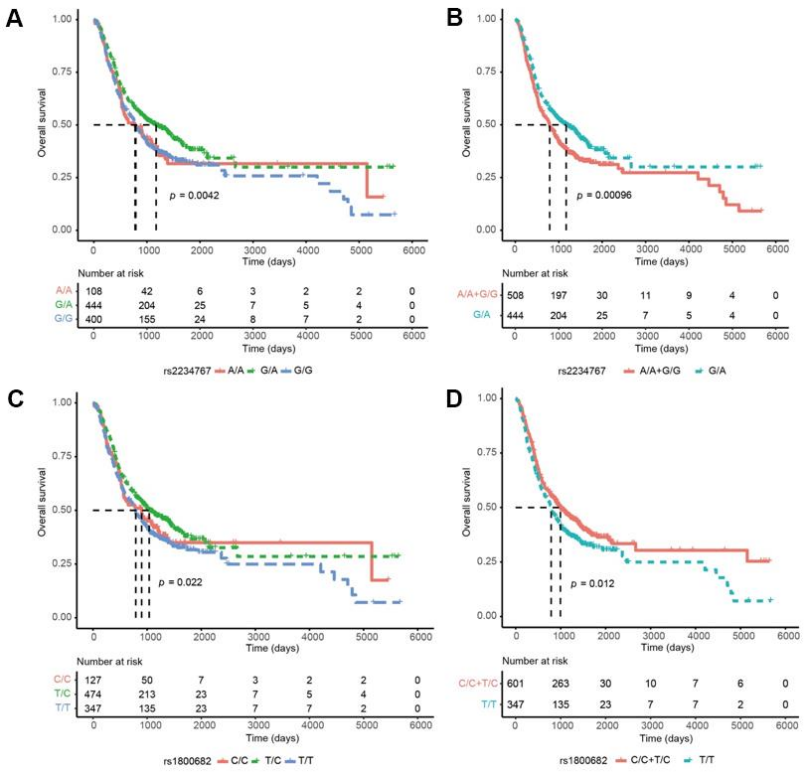
**Kaplan-Meier curves of disease-free survival (DFS) in esophageal squamous cell carcinoma (ESCC) based on genotypes, prognostic significance was found for the FAS/ACTA2 rs2234767 G/A genotypes in ESCC:** (**A**), under a reference model; (**B**), under a dominant model; and for the FAS/ACTA2 rs1800682 T/C genotypes: (**C**), under a reference model; (**D**), under a dominant model. *P* values in the univariate Cox hazard models.

We further combined the FAS/ACTA2 rs2234767 A/A+G/G and rs1800682 C/C+T/C genotypes, which exhibited a similar prognostic risk. For DFS, patients with the rs2234767 G/A and rs1800682 C/C+T/C genotypes had a decreased risk of disease progression, compared with those carrying the rs2234767 A/A+G/G (P < 0.001) and rs1800682 T/T genotypes (P = 0.012) (Figure 1).

### A new prognostic model involving sTIL, SNPs, and clinical characteristics

A multivariate Cox regression analysis was performed on 969 subjects. Age, gender, grade of differentiation, first-line treatment method, history of tea drinking, and sTIL were significantly associated with DFS (Table 4). Specifically, a higher percentage of sTIL was associated with a better prognosis (aHR = 0.958; 95% CI = 0.949 – 0.967; P < 0.001). It should be noted that there was no significant difference in DFS for the tumor grade, first-line treatment method, and history of tea drinking with adjustment for clinical characteristics, and TNM staging (Supplementary Table 3).

**Table 4:** Univariate and multivariate cox regression analyses of basic characteristics with DFS in ESCC (N=969).

**Characteristics**	**No. of patients (%)**	**Median survival (days)**	**Univariate analysis**			**Multivariate analysis**	
			***P*-value**	**HR (95% CI)**		***P*-value**	**HR (95% CI)^a^**
sTIL, mean(range)	28.80 (3.25-69.55)		**<0.001**	0.955(0.946-0.963)		**<0.001**	0.958(0.949-0.967)
Age, mean(range)	67 (42-85)		**<0.001**	1.025(1.015-1.035)		**0.023**	1.014(1.002-1.025)
Sex							
Male	645 (66.6)	855	Ref.			Ref.	
Female	324 (33.4)	1074	**0.035**	0.875(0.773-0.991)		**0.017**	**0.756(0.601-0.951)**
First-degree family history of ESCC							
No	640 (66.0)	909	Ref.			Ref.	
Yes	299 (30.9)	918	0.550	0.962(0.849-1.091)		0.262	0.926(0.81-1.059)
Missing	30 (3.1)						
Grade of Differentiation							
Gx Grading cannot be evaluated	49 (5.1)	598	Ref.			Ref.	
G1 Highly differentiated	72 (7.4)	1614	**<0.001**	1.195(0.886-1.611)		0.837	1.034(0.754-1.418)
G2 Medium differentiation	563 (58.1)	1078		1.737(1.339-2.253)		**0.006**	1.463(1.113-1.923)
G3 Poorly differentiated	181 (18.7)	750		0.724(0.547-0.957)		0.164	0.813(0.607-1.088)
G4 Undifferentiated	63 (6.5)	514		1.12(0.91-1.378)		0.304	1.12(0.902-1.39)
Missing	41 (4.2)						
First-line treatment method							
Chemotherapy	120 (12.4)	531	Ref.			Ref.	
Surgery	468 (48.3)	1517	**<0.001**	0.454(0.356-0.578)		**<0.001**	0.588(0.449-0.77)
Radiotherapy	117 (12.1)	562		0.892(0.664-1.2)		0.809	0.961(0.695-1.329)
Combination therapy	221 (22.8)	710		0.783(0.603-1.017)		0.875	0.977(0.732-1.305)
Untreated	39 (4.0)	352		1.772(1.2-2.617)		0.109	1.485(0.915-2.409)
Missing	4 (0.4)						
BMI							
<18.5	102 (10.5)	619	Ref.			Ref.	
[18.5,24)	619 (63.9)	791	**<0.001**	0.599(0.411-0.872)		0.192	0.772(0.523-1.139)
[24,28)	219 (22.6)	1351		1.159(0.861-1.561)		0.344	1.161(0.852-1.583)
≥28	29 (3.0)	1217		1.103(0.913-1.333)		0.100	1.183(0.968-1.446)
Smoking(pack-years)							
Never	400 (41.3)	1018	Ref.			Ref.	
<30.0	224 (23.1)	786	0.141	1.006(0.876-1.154)		0.101	0.845(0.69-1.034)
≥30.0	304 (31.4)	930		0.856(0.733-0.999)		0.271	0.902(0.75-1.084)
Missing	41 (4.2)						
Alcohol consumption status							
No	466 (48.1)	879	Ref.			Ref.	
Still drinking now	464 (47.9)	936	0.487	0.943(0.799-1.113)		0.187	0.859(0.685-1.077)
Missing	39 (4.0)						
Tea drinking							
No	628 (64.8)	930	Ref.			Ref.	
Yes	303 (31.3)	844	0.430	1.051(0.929-1.189)		**0.004**	1.264(1.079-1.481)
Missing	38 (3.9)						
Times of tooth brushing daily							
≤1	774 (79.9)	912	Ref.			Ref.	
>1	169 (17.4)	950	0.983	1.002(0.862-1.164)		0.435	1.068(0.905-1.26)
Missing	26 (2.7)						
Wealth score							
Q1	299 (30.9)	781	Ref.			Ref.	
Q2	181 (18.7)	844	0.401	0.831(0.673-1.027)		0.149	0.833(0.65-1.068)
Q3	221 (22.8)	936		0.992(0.811-1.213)		0.808	0.972(0.772-1.223)
Q4	170 (17.5)	952		0.928(0.762-1.131)		0.293	0.89(0.716-1.106)
Q5	98 (10.1)	1180		1.003(0.836-1.203)		0.125	0.857(0.704-1.044)
Education level							
Illiteracy	360 (37.2)	735	Ref.			Ref.	
Primary school	368 (38.0)	986	0.073	0.828(0.638-1.074)		0.200	0.807(0.582-1.12)
Junior high school	186 (19.2)	1003		1.105(0.883-1.381)		0.510	1.088(0.847-1.399)
High school and above	55 (5.7)	1288		1(0.836-1.196)		0.579	0.946(0.778-1.15)

Abbreviations: DFS, disease-free survival; CI, confidence interval; HR, hazard ratio.

^a^with adjustment for sTIL, age, sex, grade of differentiation, first-line treatment method, BMI.

**Figure 2:**
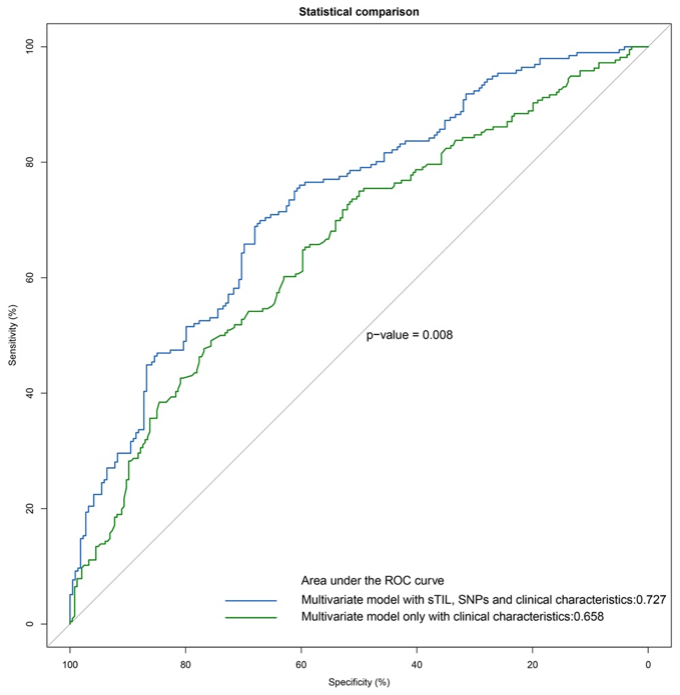
**ROC analyses of esophageal squamous cell carcinoma (ESCC) patients.** Additive prognostic value of SNPs and sTIL in patients with ESCC by comparison of area under the ROC curve (AUC) for (1) multivariate model with sTIL, SNPs and clinical characteristics, (2) multivariate model only with clinical characteristics.

Finally, we constructed a prognostic model by combining all of the independent prognostic factors (i.e., prognosis-related SNPs, sTIL) and clinical characteristics, which are statistically significant factors in a univariate analysis as shown in Table 4. The combination of prognosis-related sTIL, SNPs and clinical characteristics (AUC = 0.727) showed a significantly better prognostic value (P = 0.008) compared to the conventional clinical model (AUC = 0.658) (Figure 2).

## DISCUSSION

In this study, we found that patients with a high percentage of sTIL had a better prognosis. This finding was consistent with the results of a previous study [21, 22], in which the percentage of sTIL was estimated by pathologists. Instead, we used an automatic algorithm to calculate the percentage of sTIL, thereby ensuring the robustness and accuracy of the results.

An accurate prognostic assessment will facilitate clinical decision-making, enabling doctors to make patient-specific decisions pertaining to drug therapy, thereby improving patient prognosis [23]. We found that a model including both sTIL and the SNPs identified in our study in addition to conventional clinical factors improves the predictability of DFS. Notably, SNP rs2234767 and rs1800682 on gene *FAS/ACTA2* were found to be significantly associated with both the percentage of sTIL and patient prognosis. Several studies have described the clinical outcomes related to *FAS* SNPs in multiple types of cancer, including lung cancer and breast cancer [24–26]. By studying 338 patients with early non-small cell lung cancer (NSCLC) who underwent surgical resection, Park et al. found that the *FAS* rs1800682 T/C polymorphism may affect the survival of early NSCLC, and that the survival rate of patients with the C/C genotypes was significantly lower than those with the T/T genotype [24]. In a study of 216 women diagnosed with lymph node-positive breast cancer, Knechtel et al. found a significant association between the *FAS* rs2234767 G/A genotype (*P* = 0.040; HR = 0.451; CI = 0.496 – 1.188) with DFS in the univariate Cox regression [26] and a similar case was reported in China [25]. Further-more, a meta-analysis by Zhong et al. showed that *FAS* rs2234767 G/A were found to be associated with a decreased risk of cancer [27]. Thus, our results are consistent with these previous studies. More importantly, we are the first to show that rs1800682 and rs2234767 were also associated with the percentage of sTIL in ESCC.

Rs2234767 and rs1800682 are only significantly associated with patient survival in univariable analyses, whereas the multi-variable analysis was not significant after adjusting for sTIL, age, sex, first-line treatment method, and BMI. This implies that the two SNPs are potential confounders or the genetic variation of the gene *FAS* affect patient survival via sTIL.

*FAS*, also known as CD95, is a cell surface receptor that triggers apoptosis upon binding by its cognate ligand (FASL) as a method of maintaining immune homeostasis. *FAS* also plays a critical role in the immune-related elimination of cancer cells. It has been found that SNPs in the promoter regions of the *FAS* and *FASL* genes are associated with the differential expression of these two genes. The *FAS* rs2234767 and rs1800682 polymorphisms have been shown to reduce promoter activity and downregulate *FAS* gene expression by disrupting the *SP1* and *STAT1* transcription factor binding sites [28, 29]. In addition, these SNPs have been reported to be involved in the development of esophageal cancer, lung cancer, and breast cancer, for which apoptosis plays a key pathogenic role [30].

Furthermore, the overexpression of ANP32A was associated with aberrant expression of oncogenes and tumor suppressor, as well as immune-related pathways [31]. It is possible that individuals carrying genotype T/T of rs8030672 in gene ANP32A may be more susceptible to alterations in the state of gene modifications or DNA damage related to autoimmune regulation [20, 32, 33], when stimulated by certain carcinogen exposures such as acetaldehyde and nicotine. Subsequently, the alterations may result in reduced sTIL in the tumor microenvironment. Further studies are needed to explore the mechanism.

This study has some limitations. The SNPs we studied are derived from genes that have been shown to contribute to the risk of ESCC, however they are not comprehensive on a genome-wide level. Besides, our study only involved patients from one region. Taizhou, its population with a combination of genetic characteristics from both north and south China [34], is geographically located at the north-south border and one of the highest esophageal cancer regions in China. Therefore, our studied population is a fairly good representative of the Chinese population. We have collected information on alcohol and tea consumption, as well as individual oral health, socioeconomic status, education level, and first-line treatment, and adjusted for these factors in our analysis. However, it should still be cautious in interpreting the results, given that China is a multi-ethnic country with large geographic variation.

The findings of this study lay the groundwork for future research into the mechanisms of how sTIL and SNPs affect ESCC patient prognosis. Future research should be carried out to generalize the outcome and search for genomic variations that explain the different immune responses in ESCC and the subsequent effect on patient survival.

## CONCLUSIONS

This study aimed to investigate the association between sTIL and SNPs, and to explore their prognostic value independent of the classical clinical variables of patients with ESCC. To the best of our knowledge, this is the first study to investigate the association between SNPs of genes known to be associated with a higher risk of EC with the proportion of sTIL and their impact on patient survival. We examined the percentage of sTIL and 101 SNPs from 59 ESCC risk-related genes in 969 patients. We found that 10 SNPs from 9 genes were associated with the percentage of sTIL, and 6 SNPs were associated with prognosis.

## MATERIALS AND METHODS

### Patients and sample selection

We recruited and genotyped 1,190 EC patients between 2010 and 2013 in Taixing, China. The following inclusion criteria for cases were used: (1) age 40 – 85 years old and living in Taixing for at least five years; (2) suspected EC by endoscopy that was subsequently histopathologically confirmed. (3) newly diagnosed and newly treated with ESCC. The specific study design was as previously described [35–39]. Furthermore, 221 participants who were lost to follow-up or had low quality images from hematoxylin and eosin (H&E)-stained slides derived from tissue blocks were excluded. The patients’ personal information and clinical data (e.g., smoking, drinking, and family history) were obtained by questionnaires and patients were staged according to the 7th edition of the American Joint Committee on Cancer staging system.

### Histologic evaluation of sTIL

We used an unsupervised learning algorithm to independently perform tumor infiltrating cell detection for each analyzed image. The algorithm has been used in automated detection of tumor tissue on nuclear magnetic resonance sequences and the average MiMSeg (Mixture Model based Segmentation) has been demonstrated similar to the expert-curated decision with 89.2% consistency [40]. We scored TIL cells on the tissue slide image using the following three-step algorithm: 1) we first started with the RGB domain enhancement using a Gaussian mixture model (GMM). The GMM decomposition of signal distribution in each RGB channel was performed. The multi-class image quantization was realized based on the threshold values obtained after k-means clustering of all GMM components. An optimal number of classes was found with the use of Dunn criterion. Each level in the quantization process represents separate segments. The identified image segments were then automatically annotated into nuclei, cytoplasm, stroma and other type. 2) during the second step we focused on image segments annotated as nuclei. The lymphocyte nuclei were identified using the following features: i) the size of the lymphocyte nucleus with respect to the size of the other detected nuclei; ii) nucleus roundness; iii) nucleus eccentricity; iv) nucleus to cell size ratio; and v) nucleus staining variation. All the steps mentioned above are described in detail in Binczyk et al. [40]. The lymphocyte nucleus and cytoplasm were used to construct the lymphocytes; and 3) since stromal TILs are located either scattered or clustered in the stroma between the tumor cells but do not directly interact with tumor cells [41], identification of the tumor region is required. The percentage of sTIL’s was calculated as the ratio between the area of stromal TILs and total stroma, limited to the tumor regions.

### SNP genotyping and quality control

We selected SNPs that were previously reported to be associated with ESCC in candidate genes or GWAS studies [42, 43]. A total of 101 SNPs from 59 genes were obtained for genotyping. The SNPs were genotyped by using a three-round multiplex polymerase chain reaction procedure with next-generation sequencing technology [44].

### Follow-up

The survival time of each patient was calculated from the date of diagnosis until the date of death or until the end of the follow-up procedure period. The survival time was expressed in days.

### Statistical analysis

Distributions of continuous variables were evaluated using the Kruskal-Wallis H, and Shapiro-Wilk tests. Parametric continuous data are expressed as mean and 95% confidence intervals (95% CI). Categorical data were presented as numbers and percentages. Correlations between the genotype frequencies and clinical information were analyzed using a Chi-square test. The correlation between sTIL and SNPs was analyzed with a Kruskal-Wallis H test since the percentage of sTIL did not meet the prerequisites for parametric testing method.

Disease-free survival (DFS) was defined as the time which begins at diagnosis and up to recurrence of ESCC or the time of any death or last follow-up. Kaplan-Meier curves were displayed to evaluate long-term DFS and comparisons were performed with the log-rank test.

The Cox proportional hazard model was used for univariable analysis and multi-variable association analysis between SNPs and prognosis, adjusting for sTIL, age, sex, first-line treatment method, and BMI (i.e., confounding factors associated with ESCC survival) (Table 4). Univariate Cox regression analysis stratified by first-line treatment method was used to analyze the effect of SNPs on prognosis (Supplementary Table 1). The multivariate model with adjusting for TNM staging and other clinical characteristics was used only for the 507 patients who had clinical staging information (Supplementary Tables 2, 3). The hazard ratio (HR) and 95% confidence interval were estimated.

To investigate whether the inclusion of sTIL and SNP could improve the accuracy of a general prediction model with only clinical characteristics, we randomly divided 969 individuals into a 70% training set for training the model and a 30% test set for calculating the accuracy of the model. Receiver operating characteristic (ROC) curve was used to compare the sensitivity and specificity of each parameter for predicting disease-free survival. Area under curve (AUC) was calculated to compare model fit superiority and statistical significance was calculated by Delong’s test [45].

Statistical analyses were performed using R software (version 3.5.1, http://cran.r-project.org) and SPSS (version 21). A threshold of *P* < 0.05 was considered to be statistically significant and all tests were two-sided. Patients with missing values on any of the analyzed predictors were excluded from the univariable analysis and multivariable analyses.

### Ethical approval and consent to participate

All participants provided written informed consent. This project was approved by the institutional review boards of the School of Life Sciences, Fudan University and Qilu Hospital, Shandong University. This study was conducted in compliance with the Measures for the Administration of Human Genetic Resources issued by the Ministry of Science and Technology and the Ministry of Public Health, China, and the Declaration of Helsinki or comparable ethical standards.

### Consent for publication

Written informed consent for publication was obtained from all participants.

### Availability of supporting data

The datasets generated during and analyzed during the current study are available from the corresponding author on reasonable request.

## Supplementary Material

Supplementary Figure 1

Supplementary Table 1

Supplementary Tables 2 and 3

## References

[1] Bray F, Ferlay J, Soerjomataram I, Siegel RL, Torre LA, Jemal A. Global cancer statistics 2018: GLOBOCAN estimates of incidence and mortality worldwide for 36 cancers in 185 countries. CA Cancer J Clin. 2018; 68:394–424. 10.3322/caac.21492 30207593

[2] Zhang Y. Epidemiology of esophageal cancer. World J Gastroenterol. 2013; 19:5598–606. 10.3748/wjg.v19.i34.5598 24039351 PMC3769895

[3] Alsop BR, Sharma P. Esophageal cancer. Gastroenterol Clin North Am. 2016; 45:399–412. 10.1016/j.gtc.2016.04.001 27546839

[4] Siegel RL, Miller KD, Jemal A. Cancer statistics, 2019. CA Cancer J Clin. 2019; 69:7–34. 10.3322/caac.21551 30620402

[5] Luen SJ, Savas P, Fox SB, Salgado R, Loi S. Tumour-infiltrating lymphocytes and the emerging role of immunotherapy in breast cancer. Pathology. 2017; 49:141–55. 10.1016/j.pathol.2016.10.010 28049579

[6] Fridman WH, Pagès F, Sautès-Fridman C, Galon J. The immune contexture in human tumours: impact on clinical outcome. Nat Rev Cancer. 2012; 12:298–306. 10.1038/nrc3245 22419253

[7] Zitvogel L, Kepp O, Kroemer G. Immune parameters affecting the efficacy of chemotherapeutic regimens. Nat Rev Clin Oncol. 2011; 8:151–60. 10.1038/nrclinonc.2010.223 21364688

[8] Stoll G, Enot D, Mlecnik B, Galon J, Zitvogel L, Kroemer G. Immune-related gene signatures predict the outcome of neoadjuvant chemotherapy. Oncoimmunology. 2014; 3:e27884. 10.4161/onci.27884 24790795 PMC4004621

[9] Lee WS, Kang M, Baek JH, Lee JI, Ha SY. Clinical impact of tumor-infiltrating lymphocytes for survival in curatively resected stage IV colon cancer with isolated liver or lung metastasis. Ann Surg Oncol. 2013; 20:697–702. 10.1245/s10434-012-2752-1 23224827

[10] Kocián P, Šedivcová M, Drgáč J, Cerná K, Hoch J, Kodet R, Bartůňková J, Špíšek R, Fialová A. Tumor-infiltrating lymphocytes and dendritic cells in human colorectal cancer: their relationship to KRAS mutational status and disease recurrence. Hum Immunol. 2011; 72:1022–28. 10.1016/j.humimm.2011.07.312 21884745

[11] Liu H, Zhang T, Ye J, Li H, Huang J, Li X, Wu B, Huang X, Hou J. Tumor-infiltrating lymphocytes predict response to chemotherapy in patients with advance non-small cell lung cancer. Cancer Immunol Immunother. 2012; 61:1849–56. 10.1007/s00262-012-1231-7 22456757 PMC11029471

[12] Taube JM, Klein A, Brahmer JR, Xu H, Pan X, Kim JH, Chen L, Pardoll DM, Topalian SL, Anders RA. Association of PD-1, PD-1 ligands, and other features of the tumor immune microenvironment with response to anti-PD-1 therapy. Clin Cancer Res. 2014; 20:5064–74. 10.1158/1078-0432.CCR-13-3271 24714771 PMC4185001

[13] Salgado R, Denkert C, Demaria S, Sirtaine N, Klauschen F, Pruneri G, Wienert S, Van den Eynden G, Baehner FL, Penault-Llorca F, Perez EA, Thompson EA, Symmans WF, et al, and International TILs Working Group 2014. The evaluation of tumor-infiltrating lymphocytes (TILs) in breast cancer: recommendations by an international TILs working group 2014. Ann Oncol. 2015; 26:259–71. 10.1093/annonc/mdu450 25214542 PMC6267863

[14] Kojima T, Doi T. Immunotherapy for esophageal squamous cell carcinoma. Curr Oncol Rep. 2017; 19:33. 10.1007/s11912-017-0590-9 28361224 PMC5374168

[15] Arens R, Schoenberger SP. Plasticity in programming of effector and memory CD8 T-cell formation. Immunol Rev. 2010; 235:190–205. 10.1111/j.0105-2896.2010.00899.x 20536564 PMC2937176

[16] Shevach EM. Mechanisms of foxp3+ T regulatory cell-mediated suppression. Immunity. 2009; 30:636–45. 10.1016/j.immuni.2009.04.010 19464986

[17] Egan KM, Nabors LB, Olson JJ, Monteiro AN, Browning JE, Madden MH, Thompson RC. Rare TP53 genetic variant associated with glioma risk and outcome. J Med Genet. 2012; 49:420–21. 10.1136/jmedgenet-2012-100941 22706378 PMC3576847

[18] Lin WY, Camp NJ, Cannon-Albright LA, Allen-Brady K, Balasubramanian S, Reed MW, Hopper JL, Apicella C, Giles GG, Southey MC, Milne RL, Arias-Pérez JI, Menéndez-Rodríguez P, et al. A role for XRCC2 gene polymorphisms in breast cancer risk and survival. J Med Genet. 2011; 48:477–84. 10.1136/jmedgenet-2011-100018 21632523 PMC3932658

[19] Bozzetti F, Andreola S, Bertario L. Pathological features of rectal cancer after preoperative radiochemotherapy. Int J Colorectal Dis. 1998; 13:54–55. 10.1007/s003840050134 9548104

[20] Koag MC, Kou Y, Ouzon-Shubeita H, Lee S. Transition-state destabilization reveals how human DNA polymerase β proceeds across the chemically unstable lesion N7-methylguanine. Nucleic Acids Res. 2014; 42:8755–66. 10.1093/nar/gku554 24966350 PMC4117778

[21] Jiang D, Liu Y, Wang H, Wang H, Song Q, Sujie A, Huang J, Xu Y, Zeng H, Tan L, Hou Y, Xu C. Tumour infiltrating lymphocytes correlate with improved survival in patients with esophageal squamous cell carcinoma. Sci Rep. 2017; 7:44823. 10.1038/srep44823 28322245 PMC5359661

[22] Romagnoli G, Wiedermann M, Hübner F, Wenners A, Mathiak M, Röcken C, Maass N, Klapper W, Alkatout I. Morphological evaluation of tumor-infiltrating lymphocytes (TILs) to investigate invasive breast cancer immunogenicity, reveal lymphocytic networks and help relapse prediction: a retrospective study. Int J Mol Sci. 2017; 18:1936. 10.3390/ijms18091936 28885584 PMC5618585

[23] Galvan A, Colombo F, Frullanti E, Dassano A, Noci S, Wang Y, Eisen T, Matakidou A, Tomasello L, Vezzalini M, Sorio C, Dugo M, Ambrogi F, et al. Germline polymorphisms and survival of lung adenocarcinoma patients: a genome-wide study in two European patient series. Int J Cancer. 2015; 136:E262–71. 10.1002/ijc.29195 25196286

[24] Park JY, Lee WK, Jung DK, Choi JE, Park TI, Lee EB, Cho S, Park JY, Cha SI, Kim CH, Kam S, Jung TH, Jheon S. Polymorphisms in the FAS and FASL genes and survival of early stage non-small cell lung cancer. Clin Cancer Res. 2009; 15:1794–800. 10.1158/1078-0432.CCR-08-1770 19240174

[25] Hu Y, Xu Y, Wang Y, Ouyang T, Li JF, Wang TF, Fan ZQ, Fan T, Lin BY, Geng PL, Xie YT. [Prognostic role of apoptosis-related gene Fas-1377 and -670 polymorphisms in breast cancer]. Zhonghua Zhong Liu Za Zhi. 2009; 31:104–07. 19538884

[26] Knechtel G, Hofmann G, Gerger A, Renner W, Langsenlehner T, Szkandera J, Wolf G, Samonigg H, Krippl P, Langsenlehner U. Analysis of common germline polymorphisms as prognostic factors in patients with lymph node-positive breast cancer. J Cancer Res Clin Oncol. 2010; 136:1813–19. 10.1007/s00432-010-0839-2 20204402 PMC11828221

[27] Zhong-Xing Z, Yuan-Yuan M, Hai Zhen M, Jian-Gang Z, Li-Feng Z. FAS-1377 G/A (rs2234767) polymorphism and cancer susceptibility: a meta-analysis of 17,858 cases and 24,311 controls. PLoS One. 2013; 8:e73700. 10.1371/journal.pone.0073700 24014103 PMC3754923

[28] Sibley K, Rollinson S, Allan JM, Smith AG, Law GR, Roddam PL, Skibola CF, Smith MT, Morgan GJ. Functional FAS promoter polymorphisms are associated with increased risk of acute myeloid leukemia. Cancer Res. 2003; 63:4327–30. 12907599

[29] Huang QR, Morris D, Manolios N. Identification and characterization of polymorphisms in the promoter region of the human Apo-1/Fas (CD95) gene. Mol Immunol. 1997; 34:577–82. 10.1016/s0161-5890(97)00081-3 9393960

[30] Sun T, Miao X, Zhang X, Tan W, Xiong P, Lin D. Polymorphisms of death pathway genes FAS and FASL in esophageal squamous-cell carcinoma. J Natl Cancer Inst. 2004; 96:1030–36. 10.1093/jnci/djh187 15240787

[31] Huang S, Huang Z, Ma C, Luo L, Li YF, Wu YL, Ren Y, Feng C. Acidic leucine-rich nuclear phosphoprotein-32A expression contributes to adverse outcome in acute myeloid leukemia. Ann Transl Med. 2020; 8:345. 10.21037/atm.2020.02.54 32355789 PMC7186738

[32] Koag MC, Jung H, Kou Y, Lee S. Bypass of the major alkylative DNA lesion by human DNA polymerase η. Molecules. 2019; 24:3928. 10.3390/molecules24213928 31683505 PMC6864850

[33] Kou Y, Koag MC, Lee S. Structural and kinetic studies of the effect of guanine N7 alkylation and metal cofactors on DNA replication. Biochemistry. 2018; 57:5105–16. 10.1021/acs.biochem.8b00331 29957995 PMC6113087

[34] Suo C, Xu H, Khor CC, Ong RT, Sim X, Chen J, Tay WT, Sim KS, Zeng YX, Zhang X, Liu J, Tai ES, Wong TY, et al. Natural positive selection and north-south genetic diversity in East Asia. Eur J Hum Genet. 2012; 20:102–10. 10.1038/ejhg.2011.139 21792231 PMC3234507

[35] Chen X, Yuan Z, Lu M, Zhang Y, Jin L, Ye W. Poor oral health is associated with an increased risk of esophageal squamous cell carcinoma - a population-based case-control study in China. Int J Cancer. 2017; 140:626–35. 10.1002/ijc.30484 27778330

[36] Chen T, Cheng H, Chen X, Yuan Z, Yang X, Zhuang M, Lu M, Jin L, Ye W. Family history of esophageal cancer increases the risk of esophageal squamous cell carcinoma. Sci Rep. 2015; 5:16038. 10.1038/srep16038 26526791 PMC4630623

[37] Yang X, Chen X, Zhuang M, Yuan Z, Nie S, Lu M, Jin L, Ye W. Smoking and alcohol drinking in relation to the risk of esophageal squamous cell carcinoma: a population-based case-control study in China. Sci Rep. 2017; 7:17249. 10.1038/s41598-017-17617-2 29222520 PMC5722909

[38] Suo C, Yang Y, Yuan Z, Zhang T, Yang X, Qing T, Gao P, Shi L, Fan M, Cheng H, Lu M, Jin L, Chen X, Ye W. Alcohol intake interacts with functional genetic polymorphisms of aldehyde dehydrogenase (ALDH2) and alcohol dehydrogenase (ADH) to increase esophageal squamous cell cancer risk. J Thorac Oncol. 2019; 14:712–25. 10.1016/j.jtho.2018.12.023 30639619

[39] Suo C, Qing T, Liu Z, Yang X, Yuan Z, Yang YJ, Fan M, Zhang T, Lu M, Jin L, Chen X, Ye W. Differential cumulative risk of genetic polymorphisms in familial and nonfamilial esophageal squamous cell carcinoma. Cancer Epidemiol Biomarkers Prev. 2019; 28:2014–21. 10.1158/1055-9965.EPI-19-0484 31562207

[40] Binczyk F, Stjelties B, Weber C, Goetz M, Meier-Hein K, Meinzer HP, Bobek-Billewicz B, Tarnawski R, Polanska J. MiMSeg - an algorithm for automated detection of tumor tissue on NMR apparent diffusion coefficient maps. Inf Sci. 2017; 384:235–48. 10.1016/j.ins.2016.07.052

[41] Khoury T, Nagrale V, Opyrchal M, Peng X, Wang D, Yao S. Prognostic significance of stromal versus intratumoral infiltrating lymphocytes in different subtypes of breast cancer treated with cytotoxic neoadjuvant chemotherapy. Appl Immunohistochem Mol Morphol. 2018; 26:523–32. 10.1097/PAI.0000000000000466 28187033 PMC5550367

[42] Wu C, Kraft P, Zhai K, Chang J, Wang Z, Li Y, Hu Z, He Z, Jia W, Abnet CC, Liang L, Hu N, Miao X, et al. Genome-wide association analyses of esophageal squamous cell carcinoma in Chinese identify multiple susceptibility loci and gene-environment interactions. Nat Genet. 2012; 44:1090–97. 10.1038/ng.2411 22960999

[43] Cui R, Kamatani Y, Takahashi A, Usami M, Hosono N, Kawaguchi T, Tsunoda T, Kamatani N, Kubo M, Nakamura Y, Matsuda K. Functional variants in ADH1B and ALDH2 coupled with alcohol and smoking synergistically enhance esophageal cancer risk. Gastroenterology. 2009; 137:1768–75. 10.1053/j.gastro.2009.07.070 19698717

[44] Chen K, Zhou YX, Li K, Qi LX, Zhang QF, Wang MC, Xiao JH. A novel three-round multiplex PCR for SNP genotyping with next generation sequencing. Anal Bioanal Chem. 2016; 408:4371–77. 10.1007/s00216-016-9536-6 27113460

[45] DeLong ER, DeLong DM, Clarke-Pearson DL. Comparing the areas under two or more correlated receiver operating characteristic curves: a nonparametric approach. Biometrics. 1988; 44:837–45. 10.2307/2531595 3203132

