# Supplementary material for: Tumor infiltrating lymphocyte signature is associated with single nucleotide polymorphisms and predicts survival in esophageal squamous cell carcinoma patients
Source: Aging (Albany NY). 2021 Apr 4;13(7):10369–86. doi: 10.18632/aging.202798 (PMC8064198; doi:10.18632/aging.202798)
Supplement: Supplementary Figure 1 [file aging-13-202798-s001.pdf]

SUPPLEMENTARY FIGURES

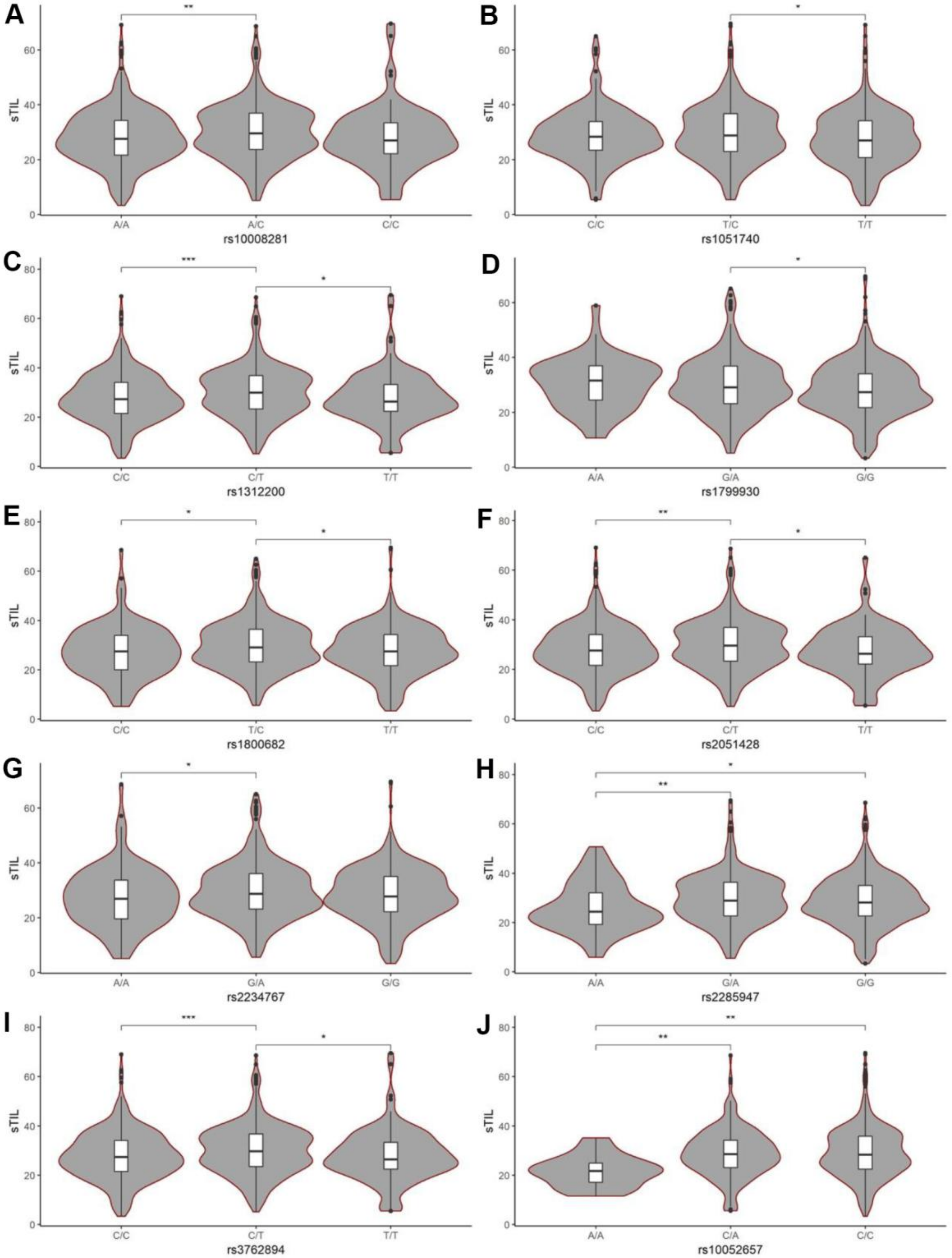

Supplementary Figure 1. Comparison of sTIL between different genotypes of 10 SNPs with significant differences. (**A**–**J**) The vertical axis represents the percentage of sTIL and the horizontal axis is the genotypes of each SNP. * P < 0.05; ** P < 0.01; *** P < 0.001.
