# Supplementary material for: Tumor infiltrating lymphocyte signature is associated with single nucleotide polymorphisms and predicts survival in esophageal squamous cell carcinoma patients
Source: Aging (Albany NY). 2021 Apr 4;13(7):10369–86. doi: 10.18632/aging.202798 (PMC8064198; doi:10.18632/aging.202798)
Supplement: Supplementary Table 1 [file aging-13-202798-s002.doc]

**Supplementary Table 1.** Univariate Cox regression analyses of SNPs for DFS stratified by first-line treatment (N=969).

| **SNP** | **A/B**  **alleles** | **Chemotherapy** | | | **Combition therapy** | | | **Radiotherapy** | | | **Surgery** | | |
| --- | --- | --- | --- | --- | --- | --- | --- | --- | --- | --- | --- | --- | --- |
|  |  | **Count (%)** | **P-value** | **HR (95% CI)** | **Count (%)** | **P-value** | **HR (95% CI)** | **Count (%)** | **P-value** | **HR (95% CI)** | **Count (%)** | **P-value** | **HR (95% CI)** |
| rs1801131 | A/A | 78 (70.3) | 0.863 | Ref. | 147 (70.0) | 0.14 | Ref. | 77 (69.4) | 0.303 | Ref. | 307 (69.0) | **0.002** | Ref. |
|  | A/C | 32 (28.8) |  | 0.898(0.555-1.454) | 59 (28.1) |  | 1.439(1.003-2.064) | 31 (27.9) |  | 1.33(0.813-2.178) | 119 (26.7) |  | 1.215(0.9-1.639) |
|  | C/C | 1 ( 0.9) |  | 0.7(0.097-5.068) | 4 ( 1.9) |  | 1.208(0.359-4.062) | 3 ( 2.7) |  | 0.38(0.053-2.753) | 19 ( 4.3) |  | 2.52(1.478-4.298) |
| rs994771 | C/C | 13 (11.0) | 0.759 | Ref. | 30 (13.8) | 0.257 | Ref. | 15 (13.2) | 0.151 | Ref. | 43 ( 9.4) | 0.746 | Ref. |
|  | T/C | 47 (39.8) |  | 1.121(0.57-2.206) | 94 (43.1) |  | 0.71(0.434-1.161) | 39 (34.2) |  | 0.627(0.324-1.213) | 184 (40.3) |  | 0.851(0.537-1.347) |
|  | T/T | 58 (49.2) |  | 0.947(0.486-1.844) | 94 (43.1) |  | 0.664(0.406-1.086) | 60 (52.6) |  | 0.535(0.285-1.005) | 230 (50.3) |  | 0.841(0.536-1.319) |
| rs2234767 | A/A | 17 (14.3) | 0.284 | Ref. | 21 ( 9.7) | 0.506 | Ref. | 11 ( 9.6) | 0.41 | Ref. | 55 (12.0) | **0.041** | Ref. |
|  | G/A | 47 (39.5) |  | 0.658(0.359-1.209) | 109 (50.2) |  | 0.762(0.439-1.321) | 54 (47.0) |  | 1.101(0.49-2.477) | 226 (49.2) |  | 0.753(0.494-1.148) |
|  | G/G | 55 (46.2) |  | 0.621(0.34-1.134) | 87 (40.1) |  | 0.897(0.515-1.563) | 50 (43.5) |  | 1.457(0.648-3.272) | 178 (38.8) |  | 1.075(0.706-1.635) |
| rs1800682 | C/C | 19 (16.2) | 0.118 | Ref. | 25 (11.5) | 0.621 | Ref. | 12 (10.4) | 0.831 | Ref. | 66 (14.5) | **0.078** | Ref. |
|  | T/C | 53 (45.3) |  | 0.624(0.352-1.107) | 114 (52.3) |  | 0.862(0.512-1.451) | 61 (53.0) |  | 1.032(0.484-2.201) | 239 (52.4) |  | 0.924(0.615-1.386) |
|  | T/T | 45 (38.5) |  | 0.534(0.293-0.975) | 79 (36.2) |  | 1.02(0.598-1.74) | 42 (36.5) |  | 1.181(0.541-2.578) | 151 (33.1) |  | 1.277(0.843-1.934) |
| rs8030672 | T/A | 6 ( 5.0) | 0.086 | Ref. | 19 ( 8.6) | 0.359 | Ref. | 5 ( 4.3) | 0.084 | Ref. | 30 ( 6.4) | **0.078** | Ref. |
|  | T/T | 114 (95.0) |  | 3.415(0.839-13.905) | 202 (91.4) |  | 0.766(0.432-1.355) | 111 (95.7) |  | 5.703(0.793-41.021) | 437 (93.6) |  | 1.769(0.938-3.337) |
| rs25487 | A/A | 5 ( 4.2) | **0.007** | Ref. | 9 ( 4.1) | 0.397 | Ref. | 8 ( 7.0) | 0.306 | Ref. | 20 ( 4.4) | 0.071 | Ref. |
|  | G/A | 37 (31.4) |  | 2.39(0.814-7.02) | 94 (42.7) |  | 0.98(0.449-2.14) | 46 (40.0) |  | 0.595(0.262-1.353) | 163 (35.7) |  | 0.89(0.487-1.627) |
|  | G/G | 76 (64.4) |  | 1.206(0.424-3.433) | 117 (53.2) |  | 0.782(0.359-1.703) | 61 (53.0) |  | 0.533(0.238-1.19) | 273 (59.9) |  | 0.664(0.367-1.202) |

Abbreviations: DFS, disease-free survival; CI, confidence interval; HR, hazard ratio.
