# Supplementary material for: Tumor infiltrating lymphocyte signature is associated with single nucleotide polymorphisms and predicts survival in esophageal squamous cell carcinoma patients
Source: Aging (Albany NY). 2021 Apr 4;13(7):10369–86. doi: 10.18632/aging.202798 (PMC8064198; doi:10.18632/aging.202798)
Supplement: Supplementary Tables 2 and 3 [file aging-13-202798-s003.pdf]

SUPPLEMENTARY TABLES

Supplementary Table 2. Univariate and multivariate cox regression analyses of SNPs for DFS (N=507).

SNP	Position	Gene (or the nearest)	A/B alleles	Count	Median survival (days)	Univariate analysis		Multivariate analysis	
						P-value	HR (95% CI)	*P*-value	HR (95% CI)
rs1801131	chr1:11794419	MTHFR	A/A	338	1172	0.230	Ref.		
			A/C	123	1051		1.059(0.8-1.402)	0.497	1.105 (0.829-1.472) [a]
			C/C	18	563		1.635(0.929-2.876)	**0.023**	1.947(1.098-3.453) [a]
rs994771	chr4:99406646	LOC102723576	C/C	53	768	0.480	Ref.		
			T/C	200	1033		0.828(0.558-1.228)	0.406	0.844(0.566-1.259) [a]
			T/T	240	1225		0.787(0.534-1.16)	0.246	0.793(0.535-1.174) [a]
rs2234767	chr10:88989499	FAS	A/A	59	896	**0.018**	Ref.		
		ACTA2	G/A	254	1482		0.746(0.511-1.09)	**0.025**	0.640(0.434-0.945) [b]
			G/G	184	855		1.067(0.727-1.566)	0.829	0.957(0.645-1.421) [b]
rs1800682	chr10:88990206	FAS	C/C	70	1092	**0.020**	Ref.		
		ACTA2	T/C	269	1424		0.825(0.575-1.182)	0.064	0.706(0.488-1.021) [b]
			T/T	157	837		1.192(0.82-1.732)	0.911	1.022(0.693-1.509) [b]
rs8030672	chr15:68766745	ANP32A	T/A	30	1734	0.085	Ref.		
			T/T	475	1014		1.663(0.932-2.969)	0.318	1.350 (0.749-2.433) [a]
rs25487	chr19:43551574	XRCC1	A/A	21	883	0.107	Ref.		
			G/A	185	801		0.875(0.502-1.527)	0.389	0.779(0.442-1.375) [a]
			G/G	290	1378		0.692(0.4-1.197)	0.057	0.578(0.328-1.017) [a]

Abbreviations: DFS, disease-free survival; CI, confidence interval; HR, hazard ratio.
[a]with adjustment for sTIL, age, sex, TNM stage, grade of differentiation, first-line treatment method, BMI.
[b]with adjustment for age, sex, TNM stage, grade of differentiation, first-line treatment method, BMI.

Supplementary Table 3. Univariate and multivariate cox regression analyses of basic characteristics with DFS in ESCC (N=507).

Characteristics	No. of patients (%)	Median survival (days)	Univariate analysis		Multivariate analysis	
			P-value	HR (95% CI)	P-value	HR (95% CI) [a]
sTIL, mean(range)	28.47 (5.13-69.04)		**<0.001**	0.954(0.942-0.966)	**<0.001**	0.958(0.945-0.971)
Age, mean(range)	66 (42-85)		**0.008**	1.019(1.005-1.034)	**0.289**	1.009(0.992-1.027)
Sex						
Male		930	Ref.		Ref.	
Female	170 (33.9)	1812	**0.005**	0.767(0.638-0.922)	**0.002**	0.566(0.398-0.805)
First-degree family history of ESCC						
No	337 (66.5)	1176	Ref.		Ref.	
Yes	152 (30.0)	972	0.639	1.044(0.871-1.252)	0.834	1.021(0.84-1.242)
Missing	18 (3.6)					
TNM stage						
0+I+II	301 (59.4)	1812				
III+IV	206 (40.6)	528	**<0.001**	2.545(2.007-3.228)	**<0.001**	2.09(1.583-2.76)
Grade of Differentiation						
Gx Grading cannot be evaluated	9 (1.8)	579	Ref.		Ref.	
G1 Highly differentiated	47 (9.3)	1812	**<0.001**	1.021(0.608-1.715)	0.243	0.718(0.412-1.252)
G2 Medium differentiation	340 (67.1)	1220		2.184(1.398-3.412)	0.262	1.33(0.808-2.191)
G3 Poorly differentiated	60 (11.8)	700		0.571(0.371-0.879)	0.181	0.725(0.453-1.161)
G4 Undifferentiated	49 (9.7)	470		1.212(0.895-1.64)	0.154	1.264(0.916-1.746)
Missing	2 (0.4)					
First-line treatment method						
Chemotherapy	59 (11.6)	520	Ref.		Ref.	
Surgery	97 (19.1)	1451	**<0.001**	0.49(0.35-0.687)	0.128	0.737(0.498-1.092)
Radiotherapy	50 (9.9)	609		0.851(0.541-1.337)	0.758	1.083(0.652-1.799)
Combination therapy	296 (58.4)	981		0.62(0.415-0.926)	0.571	0.878(0.561-1.375)
Untreated	4 (0.8)	188		3.012(0.929-9.765)	0.293	1.936(0.566-6.63)
Missing	1 (0.2)					
BMI						
<18.5	53 (10.5)	700	Ref.		Ref.	
[18.5,24)	310 (61.1)	1003	**0.046**	0.71(0.44-1.145)	0.938	0.98(0.59-1.628)
[24,28)	125 (24.7)	1812		1.189(0.81-1.746)	0.183	1.316(0.878-1.972)
≥28	19 (3.7)	1172		1.17(0.906-1.512)	0.180	1.205(0.917-1.583)
Smoking(pack-years)						
Never	213 (42)	1378	Ref.		Ref.	
<30.0	108 (21.3)	862	0.196	1.106(0.908-1.348)	0.227	0.829(0.611-1.124)
≥30.0	163 (32.1)	1120		0.845(0.674-1.059)	0.943	1.01(0.771-1.323)
Missing	23 (4.5)					
Alcohol consumption status						
No	238 (46.9)	1074	Ref.		Ref.	
Still drinking now	247 (48.7)	1109	0.87	1.02(0.802-1.298)	0.284	0.829(0.589-1.168)
Missing	22 (4.3)					
Tea dringking						
No	337 (66.5)	1176	Ref.		Ref.	
Yes	149 (29.4)	963	0.334	1.093(0.913-1.309)	0.189	1.176(0.923-1.498)
Missing	21 (4.1)					
Times of tooth brushing daily						
≤1	409 (80.7)	1014	Ref.		Ref.	
>1	81 (16.0)	1234	0.577	0.937(0.746-1.178)	0.717	0.956(0.748-1.221)
Missing	17 (3.4)					
Wealth score						
Q1	148 (29.2)	1014	Ref.		Ref.	
Q2	105 (20.7)	1009	0.703	0.882(0.657-1.186)	0.188	0.784(0.546-1.126)

Q3	98 (19.3)	2371		1.073(0.8-1.44)	0.71	1.068(0.756-1.507)
Q4	101 (19.9)	972		0.927(0.71-1.211)	0.496	0.903(0.673-1.212)
Q5	55 (10.8)	1378		0.894(0.679-1.178)	0.111	0.79(0.59-1.056)
Education level						
Illiteracy	167 (32.9)	984	Ref.		Ref.	
Primary school	191 (37.7)	1118	0.605	0.765(0.508-1.152)	0.149	0.7(0.431-1.136)
Junior high school	121 (23.9)	1109		0.915(0.652-1.283)	0.776	1.054(0.733-1.515)
High school and above	28 (5.5)			0.963(0.752-1.233)	0.522	0.917(0.702-1.197)

Abbreviations: DFS, disease-free survival; CI, confidence interval; HR, hazard ratio.

[a]with adjustment for sTIL, age, sex, TNM stage, grade of differentiation, first-line treatment method, BMI.
